# HTLV-1 Alters T Cells for Viral Persistence and Transmission

**DOI:** 10.3389/fmicb.2018.00461

**Published:** 2018-03-20

**Authors:** Azusa Tanaka, Masao Matsuoka

**Affiliations:** ^1^Department of Drug Discovery Medicine, Graduate School of Medicine, Kyoto University, Kyoto, Japan; ^2^Department of Hematology, Rheumatology and Infectious Diseases, Faculty of Life Sciences, Kumamoto University, Kumamoto, Japan; ^3^Institute for Frontier Life and Medical Sciences, Kyoto University, Kyoto, Japan

**Keywords:** HTLV-1, HBZ, tax, viral oncogenesis, Regulatory T Cell

## Abstract

Human T-cell leukemia virus type 1 (HTLV-1) was the first retrovirus to be discovered as a causative agent of adult T-cell leukemia-lymphoma (ATL) and chronic inflammatory diseases. Two viral factors, Tax and HTLV-1 bZIP factor (HBZ), are thought to be involved in the leukemogenesis of ATL. Tax expression is frequently lost due to DNA methylation in the promoter region, genetic changes to the *tax* gene, and deletion of the 5′ long terminal repeat (LTR) in approximately half of all ATL cases. On the other hand, *HBZ* is expressed in all ATL cases. HBZ is known to function in both protein form and mRNA form, and both forms play an important role in the oncogenic process of HTLV-1. HBZ protein has a variety of functions, including the suppression of apoptosis, the promotion of proliferation, and the impairment of anti-viral activity, through the interaction with several host cellular proteins including p300/CBP, Foxp3, and Foxo3a. These functions dramatically modify the transcriptional profiling of host T cells. *HBZ* mRNA also promotes T cell proliferation and viability. HBZ changes infected T cells to CCR4^+^TIGIT^+^CD4^+^ effector/memory T cells. This unique immunophenotype enables T cells to migrate into various organs and tissues and to survive *in vivo*. In this review, we summarize how HBZ hijacks the transcriptional networks and immune systems of host T cells to contribute to HTLV-1 pathogenesis on the basis of recent new findings about *HBZ* and *tax*.

## Introduction

The burden of viral infection in cancer is high, with estimates having more than 20% of cancer cases caused by infection (Bouvard et al., 2009). One oncogenic virus, human T-cell leukemia virus type 1 (HTLV-1), was identified in the United States and Japan almost 40 years ago (Poiesz et al., 1980; Yoshida et al., 1982). Thereafter, HTLV-1 was found to be a causative agent of adult T-cell leukemia-lymphoma (ATL) and HTLV-1-associated myelopathy/tropical spastic paraparesis (HAM/TSP) (Hinuma et al., 1981; Gessain et al., 1985; Gallo, 2005). Today, it is estimated that HTLV-1 infects approximately 10 million people worldwide (Gessain and Cassar, 2012). HTLV-1 transmits through cell-to-cell contacts (Igakura et al., 2003; Pais-Correia et al., 2010), while the free virus shows poor infectivity with the exception of dendritic cells (DCs) that can be infected without cell-to-cell contact (Derse et al., 2001; Jones et al., 2008; Mazurov et al., 2010; Alais et al., 2015). Accordingly, HTLV-1 increases its copy number primarily by triggering the proliferation of infected cells to facilitate its transmission (Etoh et al., 1997; Cavrois et al., 1998). These properties distinguish HTLV-1 from another well-known human retrovirus, human immunodeficiency virus type 1 (HIV-1). The chemokine receptors CXCR4 and CCR5 function as co-receptors for HIV-1 to infect host CD4^+^ T cells. Unlike HIV-1, HTLV-1 can infect a variety of cell types, but more than 90% of infected cells are CD4^+^ memory T cells *in vivo* (Richardson et al., 1990; Rizkallah et al., 2017).

## Expression and Function of Tax

The HTLV-1 provirus is 9 kb in length and contains multiple coding regions for Gag, Pol, Env, p12, p30, p13, Rex, Tax, and HBZ. Among the viral proteins of HTLV-1, Tax can activate various signal pathways including the NF-κB and AP-1 pathways (Grassmann et al., 2005; Gazon et al., 2018). It also can induce T-cell leukemia or lymphoma *in vivo* (Grossman et al., 1995; Hasegawa et al., 2006). However, Tax expression is frequently undetectable in ATL cases due to genetic and epigenetic aberrations (Takeda et al., 2004). Importantly, nonsense mutations in the *tax* gene are often observed not only in ATL cases but also in infected cells of asymptomatic HTLV-1 carriers (Furukawa et al., 2001; Fan et al., 2010). Tax was initially discovered as the viral trans-activator for HTLV-1 RNA transcription from a promoter located in the 5′ LTR (Felber et al., 1985), thus its expression is essential for viral replication. However, it is a major target antigen recognized by cytotoxic T lymphocytes (CTL) (Kannagi et al., 1991). Therefore, Tax expression is tightly controlled for the survival of HTLV-1 infected cells in order to evade host immunosurveillance. Tax expression is usually silenced in ATL cells, but a single cell transcript analysis has revealed that Tax expression is not completely suppressed and that a small percentage of MT-1 cells transiently express Tax (Mahgoub et al., 2018). Because Tax expression is necessary for *de novo* infection, it may play a key role in the spreading of HTLV-1. Taken together, Tax expression is usually suppressed in order to escape from CTL, but at the same time, Tax is transiently expressed to maintain and expand HTLV-1 infected cells. These findings suggest that another key regulator may contribute to the onset of ATL.

## HBZ and Its Role in the Oncogenesis

The HTLV-1 bZIP factor (HBZ) was first identified in 2002 as a novel viral protein that contains a N-terminal transcription activation domain and a leucine zipper motif in its C-terminus (Gaudray et al., 2002). It has been reported that *HBZ* mRNA is expressed in all ATL cases. Its mRNA form promotes the proliferation of T-cells, and its protein form induces the development of T-cell lymphomas in transgenic mice, indicating that HBZ is critical for the proliferation of ATL cells and leukemogenesis (Satou et al., 2006, 2011).

## The Localization of HBZ and Its Function in the Nucleus and Cytoplasm

**FIGURE 1:**
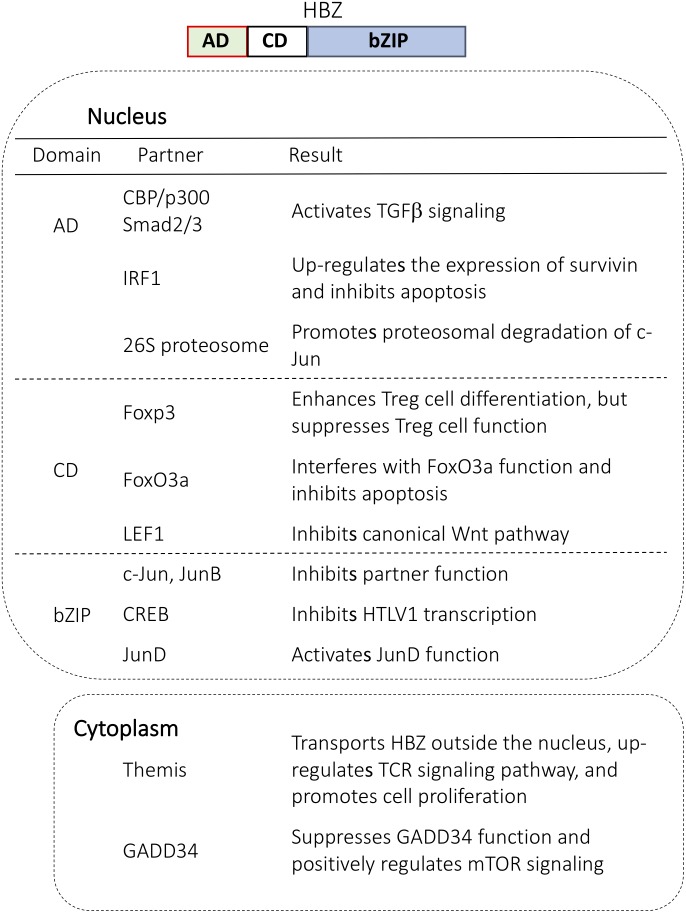
Cellular proteins that interact with HBZ in the nucleus and in the cytoplasm. HBZ has three domains: activation domain (AD), central domain (CD), and basic ZIP domain (bZIP). Each domain interacts with important regulators and modulates cellular function.

HTLV-1 bZIP factor contains nuclear localization signals in its central/bZIP domain and nuclear export signals in its N terminus (Hivin et al., 2005). HBZ is mainly localized in the cytoplasm of peripheral blood mononuclear cells (PBMCs) in HAM/TSP patients, suggesting cytoplasmic HBZ as a possible biomarker of the disorder (Baratella et al., 2017). In addition, cytoplasmic HBZ interacts with GADD34 to suppress GADD34 function and positively regulate the mechanistic target of rapamycin (mTOR) signaling pathway (Mukai and Ohshima, 2014) (**Figure 1**). Finally, the cytoplasmic localization of HBZ protein in T cells depends on the host factor THEMIS (Kinosada et al., 2017). Since THEMIS is expressed only in T cells, this function might explain why HTLV-1 promotes the proliferation of T cells.

Compared to cytoplasmic HBZ, far more studies have focused on the function of nuclear HBZ. HBZ in the nucleus makes a complex with several important transcription factors including p300, p65, LEF1, AP-1 transcription factors and forkhead family proteins (Basbous et al., 2003; Thebault et al., 2004; Satou et al., 2011; Zhao et al., 2011; Ma et al., 2013; Tanaka-Nakanishi et al., 2014) (**Figure 1**). For example, HBZ binds to p65 to diminish the p65 affinity for DNA and thus suppresses the classical pathway of NF-κB. Also, the interaction with c-Jun leads to a transcriptional repression of AP-1 regulated genes. Overall, nuclear HBZ has various functions necessary for the onset of ATL.

## Target Cells of HTLV-1 Infection

HTLV-1 provirus is mainly detected in T cells *in vivo*. In particular, most provirus (∼90%) was found in CD4^+^effector/memory T cells, while the remaining 10% of provirus was detected in CD8^+^ T cells (Yasunaga et al., 2001). HTLV-1 infected CD4^+^ T cells tend to express cell adhesion molecule 1 (CADM1), C-C chemokine receptor type 4 (CCR4), T cell immunoreceptor with Ig and ITIM domains (TIGIT), CD45RO, and sometimes CD25, indicating that these cells are effector/memory T cells (Bangham and Matsuoka, 2017). These findings suggest two scenarios for the transmission of HTLV-1. First, HTLV-1 preferentially infects these subpopulations of CD4^+^ T cells. It has been reported that Tax induces the expression of CCL22, one ligand of CCR4 (Hieshima et al., 2008), which might attract CCR4^+^ T cells and transmit the virus in the periphery. Second, HTLV-1 infection itself induces the differentiation of T cells or modifies the immunophenotype to a specific phenotype. To answer this question, we focus on HBZ below.

## HTLV-1 Infection in Hematopoietic Stem Cells

**FIGURE 2:**
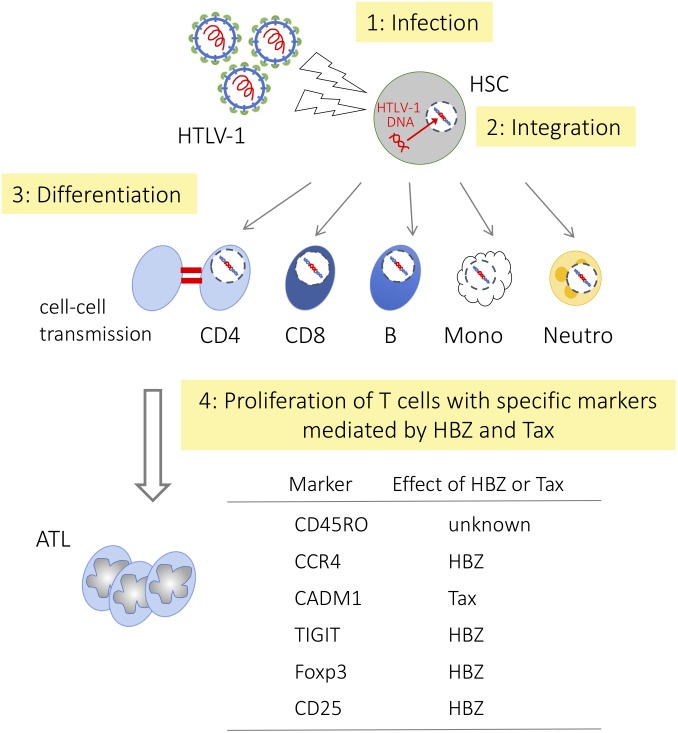
Differentiation of hematopoietic cells and HTLV-1 infection HTLV-1 infected HSCs differentiate into diverse hematopoietic cell lineages, but only memory CD4^+^ T cells become predominant. HSC, hematopoietic stem cell; CD4, CD4^+^ T cell; CD8, CD8^+^ T cell; B, B cell; Mono, monocyte; Neutro, neutrophil, respectively.

Tax is essential for *de novo* infection since transcription of the sense-strand of the provirus, which is responsible for the generation of the viral genome and viral proteins such as Gag, Env, and Pol, is Tax dependent. Tax expression in the bone marrow cells of HAM/TSP patients was reported (Levin et al., 1997), suggesting *de novo* infection occurs in this area. However, it remains unknown whether hematopoietic stem cells (HSCs) are truly infected with HTLV-1 or not. Recently, Furuta et al. (2017) reported that identical integration sites were detected in multiple hematopoietic lineage cells. This result indicates that HTLV-1 infects HSCs *in vivo* and that HTLV-1 infected HSCs differentiate into diverse hematopoietic cell lineages (**Figure 2**). Since HTLV-1 infected cells possess a similar immunophenotype, viral factors are implicated to acquire the phenotype. Because HBZ is the only viral gene that is consistently expressed in infected cells and ATL cells, we consider its role.

## HBZ Induces Effector/Memory Phenotype

Here we summarize how HBZ influences hematopoietic cell differentiation to effector/memory CD4^+^ T cells by modulating the function of key regulators (**Figure 2**).

### Foxp3

As mentioned above, although HTLV-1 can infect many types of cells including HSCs, most ATL cells and HTLV-1 infected cells are CD4^+^CD45RO^+^ conventional memory T cells. It is speculated that HBZ influences the differentiation of host T cells. For instance, HBZ affects the expression and function of Foxp3, a critical regulator for the development of Treg cells. In HBZ-Tg mice, the number of CD4^+^Foxp3^+^ T cells is increased, suggesting that HBZ induces Foxp3 expression (Satou et al., 2011). HBZ promotes Foxp3 expression by activating the TGF-β/Smad pathway (Zhao et al., 2011). Although HBZ induces Foxp3 expression, HBZ directly interacts with Foxp3 to interfere with Foxp3 DNA binding activity and function. In addition, HBZ increases the number of induced Treg cells, in which Foxp3 expression is unstable, and converts them to Foxp3^-^ T cells with increased interferon-γ production (Yamamoto-Taguchi et al., 2013). These results suggest that HBZ induces Foxp3^+^ T cells but hijacks their transcriptional network, which leads to inflammatory disease in the host.

### CCR4

It is well known that CCR4 is a hallmark of ATL cells (Yoshie et al., 2002; Ishida et al., 2003; Iqbal et al., 2010). CCR4 is not only a marker of Th2 cells, but it is also involved in the suppressive function of Treg cells (Yuan et al., 2007). CCR4 mutations were frequently observed in ATL samples, with most mutations being nonsense or frame-shift (Nakagawa et al., 2014; Kataoka et al., 2015). A truncated mutant of the cytoplasmic region of CCR4 enhances PI3K/Akt signaling, the migration and proliferation of ATL cells, but still many questions remain about the mechanisms for the increased expression of CCR4 and enhanced proliferation of cells even in cells expressing wild-type CCR4. Sugata et al. (2016) studied the mechanisms and effects of CCR4 expression on ATL cells. They found that both *HBZ* RNA and HBZ protein increased the expression of CCR4 through the induction of GATA3 expression, thereby activating transcription of the *CCR4* gene promoter. Upregulated CCR4 expression is associated with enhanced T cell migration and proliferation, which are implicated in the infiltration and proliferation of HTLV-1 infected cells. They further showed that wild-type CCR4 also promotes the proliferation of T cells and that a CCR4 antagonist inhibits cell migration and proliferation effectively. Thus, CCR4 induced by HBZ is implicated in proliferation and migration of infected cells.

## TIGIT and THEMIS

One gene upregulated by HBZ is the co-inhibitory receptor T cell immunoglobulin and ITIM domain (*TIGIT*) (Yasuma et al., 2016). ATL cells and HTLV-1 infected cells highly express TIGIT on their surfaces. Enhanced expression of TIGIT induces IL-10 production, leading to the suppression of host immune responses.

Although TIGIT is a co-inhibitory receptor that suppresses T-cell activation, it does not inhibit the proliferation of ATL cells or HTLV-1 infected cells (Kinosada et al., 2017). TIGIT exerts inhibitory signal through the intracytoplasmic immunoreceptor tyrosine inhibitory motif (ITIM) that interacts with a complex of SHP-2, Grb2, and THEMIS. HBZ interacts with THEMIS and hinders the inhibitory signal from THEMIS. Cytoplasmic HBZ almost disappears after knock-down of THEMIS expression while HBZ protein exists in the nucleus, suggesting that THEMIS is responsible for cytoplasmic localization of HBZ. THEMIS is a T cell-specific protein, this finding could explain why HTLV-1 induces the proliferation of only T cells despite the fact that HTLV-1 can infect many cell types.

Taken together, these studies suggest that HTLV-1 might influence earlier stages of hematopoietic cell differentiation and induce infected HSCs or other infected progenitor cells to abnormally differentiate into CD4^+^ Treg-like cells.

## Role of Tax in T Cell Differentiation

No direct evidence has associated Tax with T-cell differentiation. Indeed, several HTLV-1 associated molecules, including Foxp3, CCR4, and TIGIT, are not induced in Tax transgenic mice, indicating that HBZ is closely linked to the expression of these molecules (Satou et al., 2011). HTLV-1 targets CD4^+^ effector/memory T cells. As described above, HBZ is mainly responsible for immunophenotypes of HTLV-1 infected cells, but Tax has also been implicated. Two T cell specific proteins, TCF-1 and LEF-1, are highly expressed in thymocytes. It has been reported that TCF1 and LEF1 perturb Tax function (Ma et al., 2015), leading to the inhibition of HTLV-1 replication in the thymus. On the other hand, TCF-1/LEF-1 expression is suppressed in peripheral T cells. Therefore, Tax is fully functional in peripheral T cells.

## Genetic Instability Induced by HBZ

Some individuals infected with HTLV-1 succumb to ATL after a long latency period (Grassmann et al., 2005). This effect suggests that along with the viral proteins Tax and HBZ, the accumulations of genetic or epigenetic alterations are required for ATL onset. Indeed, gain-of-function alterations were frequently observed in TCR/NF-κB signaling from integrated genetic and transcriptomic analysis (Kataoka et al., 2015). Recently, it has been reported that HBZ induces genomic instability in an oncogenic miRNA-dependent manner. HBZ increases the expression of miR17 and miR21, and these oncomiRs suppress the expression of *OBFC2A*, which codes a single-stranded DNA-binding protein that protects genome stability (Vernin et al., 2014).

## HBZ Interferes With the Host Transcriptional and Translational Machinery

Detailed analysis of RNA-seq data has revealed that host genes undergo frequent and provirus-dependent transcription termination. The expression level of host-genome exons located downstream of HTLV-1 was halved on average (Rosewick et al., 2017). At the same time, systematic interaction between the HTLV-1 antisense transcript corresponding to HBZ and host genes located upstream of the provirus is observed. This fusion is classified into four types depending on the location and the direction of viral insertion (genic or intergenic integration, and concordant or discordant gene-provirus orientation). In this way, HBZ transcription perturbs host gene transcription around HTLV-1 integration sites.

Both RNA and DNA viruses are heavily dependent on the host translational machinery to produce the polypeptides that are necessary for replication. Most viral mRNAs recruit 40S ribosomal subunit to initiate their translation. For example, RPS25, a component of 40S, is essential for the internal ribosome entry sites (IRESs) of hepatits C virus (Landry et al., 2009). This recruitment not only promotes viral protein synthesis, it also impedes the host innate defenses that inhibit viral protein production. It has been reported that HBZ disrupts the host translational mechanism by negatively modulating RPS25. JunD, a functional component of AP-1 has two isoforms, a full-length isoform containing 341 amino acids (JunD-FL) and a truncated isoform lacking 48 amino acids at the N terminus (ΔJunD) (Yazgan and Pfarr, 2002; Terol et al., 2017). There is a functional difference between the two isoforms. JunD-FL is the more potent transcriptional activator, but only ΔJunD induces the proliferation and transformation of cells. HBZ induces the loss of RPS25 protein, which leads to the down-regulation of JunD-FL and up-regulation of ΔJunD. As a result, HBZ shifts the function of JunD from growth suppressor to tumor promoter, a condition advantageous for HTLV-1 infected cells to proliferate. Thus, HBZ influences host transcriptional and translational machinery.

## HBZ Suppresses Apoptosis

To increase the number of infected cells, both HBZ and Tax play a key role in maintaining clonal longevity. One mechanism for this maintenance is achieved by inhibiting the apoptosis of HTLV-1 infected cells. HBZ protein perturbs the localization and function of FOXO3a to down-regulate the pro-apoptotic genes *Bim* and *FasL* (Tanaka-Nakanishi et al., 2014). Similarly, the N-terminal region of HBZ interacts with IRF-1 to inhibit IRF-1 DNA binding/transcriptional activity and reduce the number of cells undergoing apoptosis (Chopra et al., 1990; Panfil et al., 2016). Finally, in addition to HBZ protein, *HBZ* mRNA inhibits apoptosis by upregulating the expression of survivin by enhancing its promoter activity (Mitobe et al., 2015).

## Conclusion and Future Direction

HTLV-1 bZIP factor affects many aspects of host immunity to promote the survival and proliferation of the infected cells through its mRNA form and protein form. In this review, we mainly focused on recent findings about the functions of HBZ. As described above, HBZ positively or negatively regulates various host transcription factors, modulates multiple signaling pathways and perturbs T cell function and differentiation. One of the characteristics of HBZ is that it functions both in protein form and RNA form, both of which play crucial roles in cell proliferation and cell survival. Additionally, HBZ activates oncomiRs to induce genetic instability, a hallmark of cancer.

Owing to the effort of many researchers around the world, the mystery of HBZ has been gradually uncovered. Yet, it is still unknown why most ATL cells are CD4 positive memory T cells and whether HBZ contributes to this phenomenon. Answering these questions will contribute to new therapeutic targets.

## Author Contributions

All authors listed have made a substantial, direct and intellectual contribution to the work, and approved it for publication.

## Conflict of Interest Statement

The authors declare that the research was conducted in the absence of any commercial or financial relationships that could be construed as a potential conflict of interest.
